# The Role of the E2F Transcription Factor Family in UV-Induced Apoptosis

**DOI:** 10.3390/ijms12128947

**Published:** 2011-12-06

**Authors:** Mehlika Hazar-Rethinam, Liliana Endo-Munoz, Orla Gannon, Nicholas Saunders

**Affiliations:** 1 Epithelial Pathobiology Group, University of Queensland Diamantina Institute, Princess Alexandra Hospital, Queensland 4102, Australia; E-Mails: m.rethinam@uq.edu.au (M.H.-R.); l.munoz@uq.edu.au (L.E.-M.); o.gannon@uq.edu.au (O.G.); 2 School of Biomedical Sciences, University of Queensland, Queensland 4072, Australia

**Keywords:** UV, sunburn cells, E2F, apoptosis

## Abstract

The E2F transcription factor family is traditionally associated with cell cycle control. However, recent data has shown that activating E2Fs (E2F1-3a) are potent activators of apoptosis. In contrast, the recently cloned inhibitory E2Fs (E2F7 and 8) appear to antagonize E2F-induced cell death. In this review we will discuss (i) the potential role of E2Fs in UV-induced cell death and (ii) the implications of this to the development of UV-induced cutaneous malignancies.

## 1. What is UV?

Life on earth is dependent upon UV radiation as an energy source. Ironically, whilst humans are dependent upon UV radiation for their existence, UV radiation is a common and potent carcinogen for people of Caucasian descent [1].

*Ultraviolet* (beyond violet) refers to wavelengths shorter than visible violet light and longer than X-rays [2]. The UV radiation spectrum is grouped into three categories based on wavelength. UVC (200–280 nm) is the most potent carcinogenic band of UV but poses little threat to terrestrial organisms since it is almost completely absorbed by the earth’s atmosphere. Only 10% of incident UVB (280–320 nm) radiation penetrates the atmosphere. The vast majority of incident UV (greater than 90%) radiation comes from UVA (320–400 nm). Although UVA radiation predominates at sea level, UVB has the highest energy and is 1,000 times more erythematogenic than UVA [2]. Thus, the carcinogenic potential of the UV spectrum reaching the earth’s surface is a composite of a small amount of high energy UVB and a large amount of low energy UVA. Combined, UVA and UVB radiation damage DNA, disrupt pro-apoptotic signaling pathways and suppress immune responses ultimately contributing to the carcinogenic action of sunlight [3]. Despite the more potent carcinogenic activity of UVB it is only capable of penetrating the more superficial epidermal layers whereas UVA can penetrate deeper into the dermis [2].

## 2. Mutagenic Effects of UV Radiation

UV light is a physical mutagen and can ionize molecules resulting in the conversion of absorbed light energy into biochemical reactions. DNA is one of the major molecules capable of absorbing UV radiation. Absorbed UV radiation causes DNA damage via the formation of DNA lesions often referred to as photolesions [4]. DNA damage caused by UVA and UVB can be direct or indirect. Direct absorption of UVB by DNA results in the formation of photolesions such as cyclo-butanepyrimidine dimers (CPDs) and pyrimidine (6–4) pyrimidone dimers [5] (Table 1). If these pyrimidine dimers are not repaired by DNA repair mechanisms, it may result in heritable base transitions. Formation of these, C→T single or CC→TT double, transitions at dipyrimidine sites is mutagenic and the nature and the presence of these lesions are frequently referred to as the UVB signature [6]. On the other hand, UVA is not absorbed by DNA and causes DNA damage via an indirect mechanism involving the generation of reactive oxygen species (ROS) generated by UVA-mediated activation of photosensitizers (e.g., riboflavin, porphyrins, quinines) resulting in the accumulation of CPDs [7]. UVB may also cause the accumulation of ROS and hence can also facilitate indirect DNA damage, albeit to a lesser extent than observed with UVA [8] (Table 1).

## 3. Sunburn Cells (UV-Induced Cell Death)

**Figure 1:**
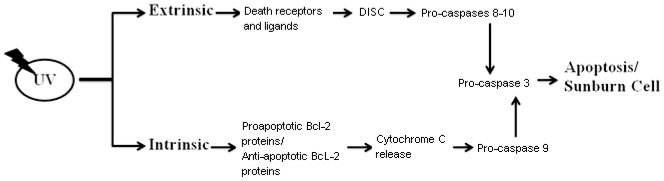
UV-mediated keratinocyte apoptosis can be initiated by extrinsic or intrinsic pathways. Extrinsic pathways include death receptor activation via death ligand binding, DISC formation, activation of pro-caspases and activation of effector caspase-3 leading to apoptosis. Activation of intrinsic pathways induces cytochrome c release from mitochondria and activation of pro-apoptotic Bcl-2 proteins and inhibition of anti-apoptotic Bcl-2 proteins, activation of pro-caspase-9 and activation of effector caspase-3 leading to apoptosis.

Following UV exposure keratinocytes will follow one of two fates. If the damage to DNA is perceived to be reparable, the keratinocytes will undergo a reversible growth arrest accompanied by the mobilisation and activation of the nucleotide excision repair system (NER). This leads to the repair of damaged DNA (mutations/DNA lesions) and is facilitated by secreted cytokines, IL12 and IL18, which can restore immune responses and prevent from UV-induced immunosuppression [9–11]. Alternatively, if the DNA damage is perceived to be too great and the cells lack the capacity to repair the damage then the cells will be induced to apoptose [12,13]. Apoptosis, or programmed cell death, is a mechanism that prevents cells from passing on mutated DNA to their progeny. Thus, the apoptotic machinery provides a means by which mutated, potentially premalignant cells are able to be eliminated [14]. UV-induced apoptosis results in the formation of so-called “sunburn cells” or apoptotic keratinocytes. Sunburn cells are easily identified by the presence of photo lesions, pyknotic nuclei and cytoplasmic shrinkage characteristic of apoptotic cells [15]. UV-induced apoptotic responses are mediated via extrinsic/death receptor signaling and intrinsic/mitochondrial death pathways [11,16]. The extrinsic death pathway is initiated by the binding of membrane death receptors TNF-R1, CD95, TRAIL-R1 and TRAIL-R2 to their cognate ligands, TNF-α, CD95L/FASL or TRAIL (TNF-related apoptosis-inducing ligand). UV can also activate CD95 death receptor signaling pathways independent of its natural ligand CD95L [17]. The subsequent formation of a death-inducing signaling complex (DISC) is characteristic of death receptor-mediated apoptosis in response to UV radiation [18,19]. Activation of death receptor signaling ultimately activates the initiator pro-caspases-8/-10 leading to the eventual activation of downstream effector procaspases-3,-6,-7 [12]. Activation of the intrinsic apoptotic pathway is stimulated by the release of cytochrome c from outer mitochondrial membrane [20]. Activation of the intrinsic apoptotic pathway is controlled by the balance between pro-apoptotic (Bax, Bak, Bad, Bid, Bim) and anti-apoptotic (Bcl-2, Bcl-Xl, Mcl-Xl) Bcl-2 family proteins. When pro-apoptotic stimuli predominate, it leads to the permeabilisation of the mitochondrial outer membrane potential leading to cytochrome C release and eventual procaspase-9 activation [21,22] (Figure 1).

## 4. Role of UV in Skin Carcinogenesis

Skin cancers are frequently divided into melanoma and non-melanoma skin cancers (NMSC). Regardless of classification, the main contributory factor in the development of cutaneous malignancies, in humans, is UV exposure [23]. Melanoma is a common and aggressive tumour type derived from melanocytes. The major forms of non-melanoma skin cancer are basal cell carcinoma (BCC) and squamous cell carcinoma (SCC) [24]. In a recent study, Trakatelli *et al.* [25] showed that NMSC had significantly increased in incidence in Caucasians in the last decade. NMSC skin cancers are the most common malignancy in Caucasians and their incidence reflects the potent carcinogenic activity of UV radiation [26]. There are a number of reviews on the molecular mechanisms associated with UV-induced skin cancer and in particular we refer the reader to other articles within this issue of the journal. Of relevance to the current review are reports that UV-induced SCC formation is associated with dysregulation of the control of proliferation, differentiation and apoptosis [27–31]. Amongst these known changes it is notable that disruption of the Rb/E2F axis is over-represented. In particular, there is considerable data relating to the expression, activity and role of dysregulated E2F1 in SCC formation [26,32–35]. For example, disruption of the Rb/E2F axis is common in almost all human cancers including SCC [36]. Loss of function mutations of p53, Rb, or upstream regulators of the Rb/E2F axis such as INK4A (p16) are frequently associated with SCC and may result from mutation, deletion or promoter hypermethylation [37–39]. Moreover, SCCs are frequently associated with amplification/activation of mitogenic pathways controlled by cyclin D1, cdk4 or EGFR [31,40]. All these events are known to contribute to the dysregulation of proliferation and differentiation [31,40,41]. In addition, dysregulation of enzymes regulating oxidative stress such as GPX2 have also been shown to contribute casually to UV-induced SCC formation [25]. Dysregulation of antioxidant enzymes is known to disturb the apoptotic axis. Indeed, apoptotic regulators related to sensitivity and response to UV-induced damage are invariably targeted during keratinocyte transformation [13]. Consequently, the major safeguard that keratinocytes use to protect themselves against UV-induced mutations, namely sunburn cell formation, is compromised in keratinocytes following exposure to carcinogenic doses of UV [41]. However, the exact mechanisms by which UV-induced mutational damage contributes to the biological events controlling keratinocyte transformation and SCC progression remain unclear.

## 5. The E2F Family

The squamous differentiation program of the epidermis involves co-ordinate regulation of proliferation, differentiation and apoptosis. The barrier functions of the epidermis depend upon the integrity of this program and its ability to respond to environmental insults such as UV radiation [7,42]. The process of squamous differentiation is a tightly regulated process in which transcription factors control the differentiation program and its barrier functions [36]. Thus, it is not surprising that disruption to transcriptional control is a frequent target in oncogenesis [43]. Many transcription factors have been implicated in the control of squamous differentiation and carcinogenesis. However, the E2F family of transcription factors have emerged as pleiotropic regulators, directly controlling (i) cell proliferation, (ii) apoptosis, (iii) differentiation, (iv) DNA-damage response and DNA repair, (v) development, (vi) senescence and (vii) autophagy [28,29,44–49]. Moreover, E2Fs are also indirectly involved in modulating the activity of important cellular signaling pathways such as MAPK, p38 and PI3-K/AKT through transcriptional regulation of upstream pathway components [50,51].

E2F was first discovered as a cellular factor required for the activation of the E2 viral promoter [29,52]. This factor was later cloned and named E2F1 [43]. However, it was quickly recognised that E2F1 was just one member of, what is now, a family of 8 members, E2Fs 1–8, coding for 10 different E2F forms [53]. The role of the E2Fs is complex. Individual E2F family members can be involved in multiple cellular activities. For example, E2F1 is directly involved in the G1/S transition of the cell cycle [54], stimulating apoptosis [44,55], suppressing differentiation [35,36,56] and acting as a transducer of the DNA damage response in keratinocytes [57]. In addition, multiple E2F family members can play a role in the same cellular functions and frequently share the same E2F binding sites in gene promoters (consensus E2F binding site is TTTCGCGC). For example E2Fs 1, 2 and 3a are all involved in the transition through the G1/S phase of the cell cycle [58]. Finally, there is emerging evidence to indicate that E2F isoform specific-sites and functions may also exist. For example, in keratinocytes we recently showed that E2F7 could selectively suppress differentiation through the repression of Sp1 transcription mediated via a selective and novel E2F7-specific response element (5′-CTCCTTTCCCCCTCCCTCAT-3′) [59]. Thus, the E2F transcription factor family is involved in complex and varied roles in cellular physiology.

**Figure 2:**
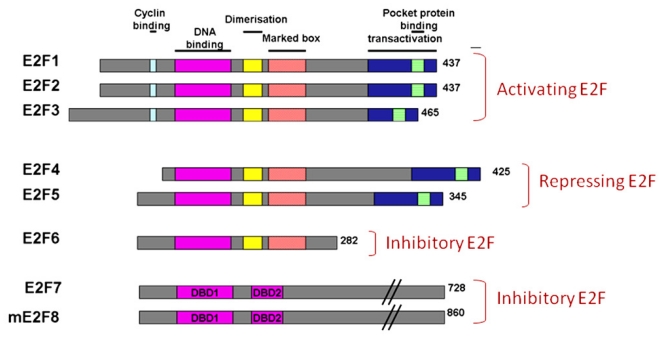
Domain organization of activating, repressive or inhibitory E2Fs. Number of amino acid is indicated on the right. Same colour boxes indicate homologues regions. There are two known E2F7 isoforms; E2F7a and E2F7b which differ only in their C termini. Both isoforms of E2F7 are expressed in all cell lines analysed.

The E2F family can be broadly classified as typical E2Fs (E2F1-6) and atypical E2Fs (E2F7-E2F8). All typical E2Fs carry one N-terminally located, evolutionary conserved, DNA-binding domain (DBD) (Figure 2). The DBD is followed by a dimerization partner binding domain [60]. There are two members of DP family proteins, DP1 and DP2 that interact with E2F isoforms through the conserved Dimerization Partner-binding domain [28]. In mammalian cells, most E2F DNA-binding takes place once E2F-DP heterodimers form [61]. In contrast, the DBD is duplicated in atypical E2Fs and they bind target gene promoters in a DP-independent fashion [28,62–66] due to the lack of a DP-binding domain. In addition, atypical E2Fs also lack a recognisable *trans*activation domain or pocket protein binding domain compared to typical E2Fs [55] (Figure 2).

E2Fs are most frequently classified based on their transcriptional activity. For example, the E2F family is generally divided into three subclasses: activator E2Fs (E2F1-E2F3a), repressor E2Fs (E2F3b-5) and inhibitory E2Fs (E2F6-E2F8). The expression of activator E2Fs varies during the cell cycle reaching a peak of activity, bound to target gene promoters via E2F response elements, during late G1/S phase. In this context, they control the expression of genes and activities required for DNA synthesis [53,67,68]. The expression and activity of the repressor E2Fs (E2F3b, E2F4, E2F5, E2F6) remain relatively constant throughout the cell cycle [53]. They bind target gene promoters during G_0_ with E2F inhibitory pocket proteins coupled with repressive histone deacetylases [39,69] and prevent promiscuous transcription of proliferation genes [53,57,70]. The so-called repressor E2Fs get their name due to their ability to actively recruit transcriptional inhibitors such as histone deacetylase 1 (e.g., E2F4 and 5) or PRC2 (E2F6) to the E2F sites resulting in transcriptional repression [57,58]. In contrast, inhibitory E2Fs (E2F 7 and 8) compete for binding sites with other E2Fs and mediate their inhibition by excluding active or repressive E2Fs from binding [55]. The expression of E2F7 and E2F8 is cell-cycle regulated. Transcription of E2F7 and E2F8 increases towards G_1_-to-S transition reaching its peak during S-to-G_2_ transition [28,57,59,60,71]. Thus, the role of E2F7/8 in cell cycle control appears to tie in with the direct inhibition of the E2F1 activities related to cell cycle traverse [42,63]. In contrast, the role of the inhibitory E2Fs in the control of differentiation appears to be isoform-specific and is mediated via isoform-specific DNA response elements [26,54]. Finally, the anti-apoptotic action of the inhibitory E2Fs appears to be mediated via direct inhibition of E2F1-mediated apoptosis [42,63]. The interplay between E2F1-stimulated apoptosis and E2F7/8-mediated inhibition of apoptosis is critical to understanding the role of E2Fs in UV-induced skin cancer formation and their potential as drugable targets for treating squamous cell carcinomas or enhancing chemotherapeutic responses.

## 6. E2F-Induced Apoptosis and Skin Cancer Formation

The ability of the different E2Fs to contribute to apoptosis especially to UV-mediated apoptosis is contentious. Much of this controversy arises from some seemingly paradoxical data relating to the action of E2F1. Earlier studies with E2F1 reported that overexpression of E2F1 in tissue culture cells and in transgenic mice caused a stimulation of apoptosis and an enhancement of tumour formation [33,34,72,73]. In particular, overexpression of E2F1 in the epidermis of transgenic mice caused elevated apoptotic indices in keratinocytes of the basal layer and an increase in skin tumour formation in mice that overexpressed E2F1 and cyclin D1 [34]. In contrast, mice transgenic for E2F4 expression in skin did not have increased apoptotic indices [74]. Similarly, mice deficient for E2F1 were predisposed to thymomas due to their inability to delete t cells via E2F1-mediated apoptosis [75,76]. These earlier studies clearly supported the concept that the pro-proliferative actions of E2F1 were oncogenic whilst the pro-apoptotic actions of E2F1 were tumour suppressive [77]. E2F1-stimulated apoptosis can be mediated by p53-dependent and p53-independent pathways. The p53-dependent pathway involves the stabilization of p53 via p14/p19^ARF^ [78] whilst activation of APAF1 and p73 or CHK2 is required for p53-independent apoptosis [79–81]. In response to UV, E2F1 transcript and protein levels increase in an ATM/ATR dependent manner and leads to accumulation of events required for apoptosis [82]. This suggested that UV-induced E2F1 mediated apoptosis in skin may have tumour suppressive effects. However, studies by Dimova and Dyson reported that the ectopic expression of E2F1 may result in the expression of survival genes suggesting that E2F1 may be anti-apoptotic under certain conditions [48]. This suggests that the role of E2F1 in regulating apoptosis may be context-specific. Consistent with this, it has been reported that E2F1 is anti-apoptotic in keratinocytes in the context of UVB irradiation [52,83]. Specifically, E2F1 deficient mice and mice transgenic for E2F1 in skin displayed increased and reduced apoptotic indices in response to UVB irradiation respectively [52,78]. Wikonkal *et al.* [52] also showed that the pro-survival effect of E2F1, in response to UVB, was p53-independent. Finally, it was shown that the pro-survival effect of E2F1, in response to UVB irradiation of keratinocytes/epidermis could be attributed to the ability of E2F1 to sense DNA damage and co-ordinate the DNA damage repair [52]. In this regard, E2F1 has been reported to function directly at sites of DNA repair to eliminate DNA photoproducts [84,85] or indirectly by controlling the transcription of genes required for DNA repair machinery [86,87].

**Figure 3:**
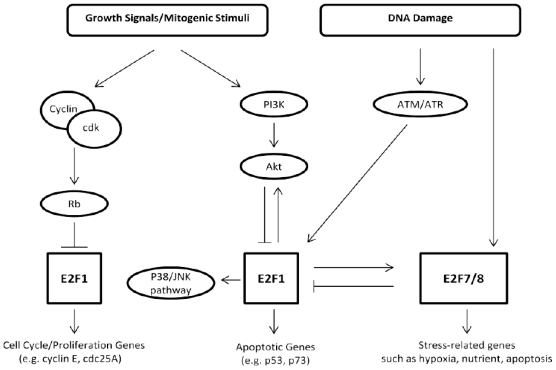
Regulatory network on E2F1 and E2F7/8 activity representing upstream events (growth-dependant and/or DNA damage mediated activation) and downstream targets.

**Figure 4:**
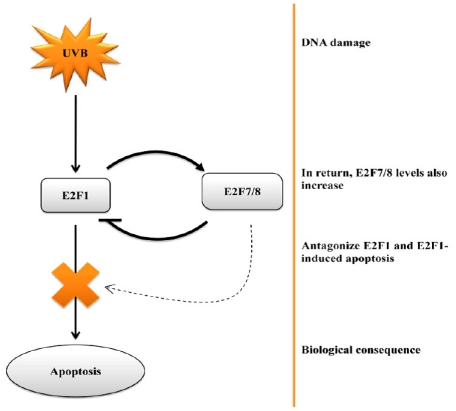
Schematic showing how E2F1 and E2F7 contribute to formation of cutaneous malignancies due to dysregulated apoptotic control.

Whilst these data appear to definitively show that E2F1 is oncogenic in skin due to its anti-apoptotic effects, there still remain some unresolved issues. For example, studies have shown that E2F1-mediated responses to UVB irradiation may be dose-dependent such that low doses of UVB activate DNA repair mechanisms whilst high doses induce apoptosis in cells in which the cellular DNA repair machinery is unable to repair the damage [88]. Moreover, another important consideration is the level of E2F1. For example, it is easy to see the benefit of a pro-survival signal being generated in response to the relatively low levels of E2F1 that may be experienced during cell cycle traverse. It is also easy to see biological justification that elevation of E2F1, in response to stressors such as UVB, could invoke apoptotic responses [89,90]. Earlier studies by Yang and his colleagues have shown that E2F6 is able to repress UV-induced apoptosis in human embryonic kidney cells via direct interaction with BRCA1 [91]. Intriguingly, the expression of E2F6 is influenced by E2F1 [92]. However, it is noteworthy that keratinocytes do not appear to express detectable levels of E2F6 [51] suggesting this situation may not apply in skin. The same cannot be said for recent studies with E2F7 and E2F8. E2F7 and 8 are inhibitory E2Fs that bind to, and repress, E2F1 transcription and E2F1-induced apoptosis [63]. Both E2F7 and E2F8 are expressed in skin [57,61] and are able to influence the cellular DNA damage response [41]. Zalmas *et al.* [41] demonstrated that DNA damage induced by etoposide treatment induced E2F7 and E2F8 expression. Moreover, they demonstrated that DNA damage invoked an increase in E2F7 and E2F8 binding to E2F-responsive genes such as E2F1 resulting in an inhibition of E2F1-mediated apoptosis [41]. In fact, microarray analysis of cells subjected to DNA damage revealed that E2F7 and E2F8 could be considered *bona fide* DNA damage response genes [58,63] (Figure 3). These studies seem to be relevant to skin UV responses since we recently reported that E2F7 plays a role in regulating proliferation, differentiation and UV-induced cytotoxicity in human keratinocytes *in vitro* [26]. Moreover, we reported that E2F1 and E2F7 were overexpressed in human squamous cell carcinomas approximately 50 fold and 200 fold respectively [26]. Such elevations in E2F1 and E2F7 are clearly pathologic and the consequences on UV-induced tumour development and progression remain unknown. However, given that E2F1 and E2F7 are said to autoregulate the expression of one another and given that E2F7 antagonises E2F1-induced apoptosis and UV-induced apoptosis in human keratinocytes [26], it would seem reasonable to speculate that E2F7 may also play a role in UV responses in human epidermis (Figure 4). Thus, apoptotic responses of keratinocytes, to UV, or chemotherapeutics, are likely to be dictated by the relative levels of E2F1 and E2F7.

In conclusion, the carcinogenic components of sunlight, relevant to humans, are UVA and UVB. UV radiation induces either a cell cycle arrest or an apoptotic response in human keratinocytes. Both the cell cycle arrest and the apoptotic response appear to be mediated by E2F1. More recently, an antagonistic form of E2F, E2F7, has been reported that antagonizes the pro-proliferative and apoptotic effects of E2F1. Both E2F1 and E2F7 are significantly overexpressed in transformed keratinocytes and there is evidence that the dysregulation of expression of the E2F1 and E2F7 isoforms may contribute to skin cancer formation.

## Figures and Tables

**Table 1:** Summary of mutagenic effects of UVA and UVB.

	UVA	UVB
Wavelength (nm)	320–400	280–320
Chromophores	Photosensitizers	DNA
Site of damage	ROS	Pyrimidine dimers (CDP) 6–4 photoproducts
Mechanism	Indirect	Direct
